# Rediscovery and phylogenetic relationships of the scolopendromorph centipede *Mimops orientalis* Kraepelin, 1903 (Chilopoda): a monotypic species of Mimopidae endemic to China, for more than one century

**DOI:** 10.3897/zookeys.932.51461

**Published:** 2020-05-12

**Authors:** Chao Jiang, Yunjun Bai, Mengxuan Shi, Juan Liu

**Affiliations:** 1 State Key Laboratory of Dao-di Herbs Breeding Base, National Resource Center for Chinese Materia Medica, China Academy of Chinese Medical Sciences, Beijing, 100700, China National Resource Center for Chinese Materia Medica, China Academy of Chinese Medical Sciences Beijing China; 2 School of Life Sciences, Henan University, Kaifeng, 475001, China Henan University Kaifeng China

**Keywords:** morphology, new distribution, Qinling Mountain

## Abstract

*Mimops orientalis* Kraepelin, 1903 is a monotypic species of Mimopidae endemic to China. The species is known only from a single specimen, the holotype. Little is known about its biology, habitat associations, or phylogenetic relationships. It was rediscovered on Qinling Mountain in Shaanxi and Henan provinces, China, 117 years after its last record. Detailed descriptions and colour photographs of living specimens are provided along with its ecology, updated conservation notes, and data on sexual dimorphism. A genetic analysis (COI, 16S rRNA, and 28S rRNA) was conducted to assess the phylogenetic relationships among Mimopidae, Cryptopidae, Scolopendridae, Scolopocryptopidae, and Plutoniumidae. The results support classifying Mimopidae as a valid family.

## Introduction

*Mimops* Kraepelin, 1903 is one of the least-known genera of scolopendromorph centipedes. The type species, *Mimops orientalis* Kraepelin, 1903, described from a single specimen from Shaanxi Province, China, has a single white ocellus on each side of the cephalic plate. Since the original description, the exact type locality of this species has been unknown, and no additional findings of *M. orientalis* have been reported. The genus *Mimops* was initially placed in the Cryptopidae and subsequently redescribed by Lewis (2006) from the holotype of *M. orientalis*, establishing a new monotypic family, Mimopidae Lewis, 2006.

However, the only material of *M. orientalis*, the unsexed holotype specimen, did not allow Lewis (2006) to clarify the behaviour or information on the distribution, ecology, habitat, or phylogenetic relationships of *Mimops*. Because *M. orientalis* possesses characteristics from the families Plutoniumidae, Cryptopidae, Scolopocryptopidae, and Scolopendridae (e.g., forcipules and coxosternal plates without teeth as in Scolopocryptopidae, ultimate legs with numerous small spines as in Cryptopidae, and unpigmented ocellus-like pale areas as in Plutoniumidae), the taxonomic status of Mimopidae is still confusing, as are the phylogenetic relationships among Mimopidae, Cryptopidae, Scolopendridae, Scolopocryptopidae, and Plutoniumidae.

Throughout 2018 and 2019, we made intensive biodiversity surveys in the Taiping National Forest Park and Longyuwan National Forest Park, China. During this fieldwork, fresh material of *M. orientalis* was collected at the edge of Qinling Mountain. It is a region that has only few previous surveys for centipedes but has been highlighted as one of 16 biodiversity hotspots (Huang et al. 2016). Our rediscovery of this species has allowed us to reassess its taxonomic status, as well as that of the family Mimopidae, and to give fuller details of this species’ morphological variations and live coloration. In this paper, we redescribe the morphological characteristics of *M. orientalis* and construct a phylogeny based on COI and 16S mitochondrial DNA fragments and 28S nuclear DNA fragments.

## Materials and methods

### Taxonomic sampling

The studied individuals of *Mimops orientalis* were collected in Taiping National Forest Park, Shaanxi, China, and Funiushan National Nature Reserve, Henan, China. Other representative samples of Cryptopidae, Scolopendridae, Scolopocryptopidae, and Plutoniumidae were collected more generally in China. During the field study, some individuals were photographed with an Olympus E-M10 II camera to record their living colour pattern. The colour description is based on that of living centipedes. Specimens are deposited in the Institute of Chinese Materia Medica, China Academy of Chinese Medical Sciences, China (**CMMI**).

The taxonomic characteristics of the specimens were observed under an Olympus SZ16 stereomicroscope. Multifocus montage images were produced using Helicon Focus 6.7.1 software from a series of source images taken by a Canon 50D digital camera attached to the stereomicroscope. Terminology applied to the external anatomy follows Bonato et al. (2010).

All newly collected centipede specimens were stored in 75% ethanol. Legs 15 and 17 of the newly collected specimens were removed and stored in 100% ethanol for DNA extraction.

### Phylogenetic sampling and DNA sequence analyses

Genomic DNA was extracted from leg tissue using a DNeasy Blood and Tissue Kit (Qiagen, Hilden, Germany). Two fragments of mitochondrial DNA (mtDNA), the encoded parts of the COI and 16S ribosomal RNA genes, and a fragment of 28S nuclear ribosomal RNA were amplified according to previously literature (Vahtera et al. 2012, 2013; Siriwut et al. 2016). PCR products were bidirectionally sequenced using an ABI 3730 DNA analyser (Applied Biosystems, CA, USA). New sequences determined in this study were aligned using ClustalW in BioEdit with default settings.

Maximum likelihood and Bayesian inference approaches were employed using MEGA X (Kumar et al. 2018) and PhyloSuite (Zhang et al. 2018), respectively, to determine the phylogenetic positions of *M. orientalis* base on the new sequences. Standard statistical tests were applied to evaluate branch support (bootstrap support and posterior probability). The best-fit substitution models and partitioning strategies were inferred for these three datasets using ModelFinder (Kalyaanamoorthy et al. 2017) integrated into PhyloSuite (Zhang et al. 2020). Based on Akaike Information Criterion, GTR+I+G4 was chosen as the best-fitting model for ML analyses and GTR+G for MrBayes analyses. Individual gene trees were constructed in MEGA using the maximum likelihood algorithm with 500 bootstrap replicates. Bayesian analyses were performed using MrBayes 3.2.6 (Ronquist et al. 2012) integrated into PhyloSuite with the default settings, and 10,000,000 MCMC generations, sampling every 100 generations. Sequences were deposited in GenBank with accessions of MT093838–MT093846, MT084401–MT084409, and MT084368–MT084375.

## Results

### Mimops orientalis

Taxon classificationAnimaliaScolopendromorphaCryptopidae

Kraepelin, 1903

365808A8-499B-568A-B375-15EFAA74A39A

#### Type material.

***Holotype***: China, Süd Schensi, August 1903, kept in Zoologisches Institut und Zoologisches Museum der Universität, Hamburg, Germany (ZMUH).

#### Type locality.

Shaanxi, China. Perhaps referring to Xi’an, Shaanxi, China.

#### Specimens examined.

Seven *Mimops orientalis* specimens were collected near a river in the Taiping National Forest Park, Hu county, Shaanxi, China (33.98N, 108.69E, 620–640 m alt.), and another *M. orientalis* were collected in 2019 from Funiushan National Nature Reserve, Luanchuan, Henan, China. *Mimops orientalis* (*n* = 8). CMMI 20190714001, adult, under a stone near a river, Longyuwan National Forest Park, Luanchuan, Henan, China, (33.713N, 111.775E, 1110 m alt.) collected by Mengxuan Shi, on 14 Jul. 2019. CMMI 20190908001, adult male, under a stone near a ditch, Taiping National Forest Park, Hu county, Shaanxi, China (33.98N, 108.69E, 630 m alt.), collected by Chao Jiang, on 08 Sept. 2019. CMMI 20190908002, juvenile, under a stone in some brushwood, Taiping National Forest Park, Hu county, Shaanxi, China (33.98N, 108.69E, 620 m alt.), collected by Chao Jiang, on 08 Sept. 2019. CMMI 20190908003 and CMMI 20190908004, juvenile, under stones in grass halfway up a steep hill, Taiping National Forest Park, Hu county, Shaanxi, China (33.98N, 108.69E, 640 m alt.), collected by Chao Jiang, on 08 Sept 2019. CMMI 20190908005, adult, under a trash bin near the road, Taiping National Forest Park, Hu county, Shaanxi, China (33.98N, 108.69E, 626 m alt.), collected by Chao Jiang, on 08 Sept. 2019. CMMI 20190908006, subadult, under a stone near the collection site of specimen CMMI 20190908005, collected by Chao Jiang, on 08 Sept. 2019. CMMI 20190908007, juvenile, under a stone of brushwood, Taiping National Forest Park, Hu county, Shaanxi, China (33.98N, 108.69E, 620 m alt.), collected by Chao Jiang, on 08 Sept 2019.

#### Other specimens for DNA sequencing.

Plutoniumidae: *Theatops chuanensis* Di et al., 2010, CMMI 20190405013, Tianzishan National Reserve, Zhangjiajie, Hunan province, China. CMMI 20190606004, Wen county, Longnan, Gansu province, China.

Cryptopidae: *Cryptops* sp., CMMI 20190413007, Longjin road, Huoshan county, Liu’an, Anhui province, China.

Scolopendridae: *Scolopendra mutilans* L. Koch, CMMI 20190331001, Qianfodong National Forest Park, Duodao distinct, Jinmen, Hubei province, China. CMMI 20190702006, Xiangshan, Dinghai distinct, Zhoushan, Zhejiang province, China.

Scolopocryptopidae: *Scolopocryptops nigrimaculatus* Song, Song & Zhu, 2004, CMMI 20181207002, Jinxi hotel, West Lake, Hangzhou, Zhejiang province, China.

#### Distribution.

**Figure 1.**
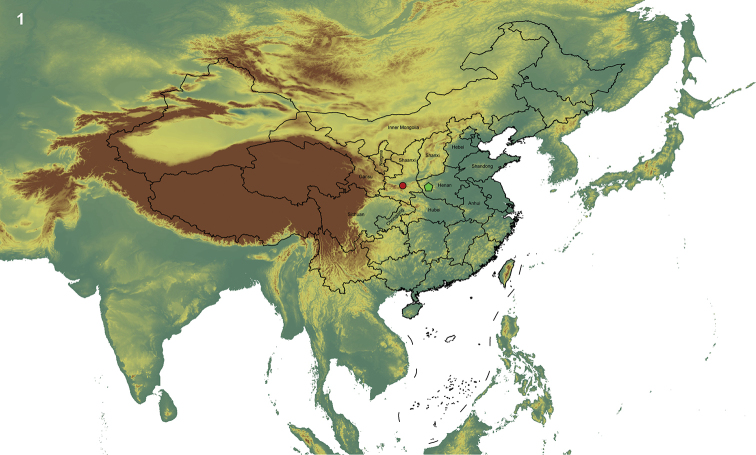
The current known distribution of *Mimops orientalis*. Two main record localities were in Shaanxi and Henan provinces, China, indicated by a red dot and a green pentagon, respectively; the red dot perhaps indicates the type locality.

Currently, we have confirmed that *M. orientalis* occurs on Qinling Mountain (Shaanxi and Henan provinces) and inhabits forests near rivers above 500–1200 m elevation (Fig. 1).

#### Ecology.

**Figures 2–5.**
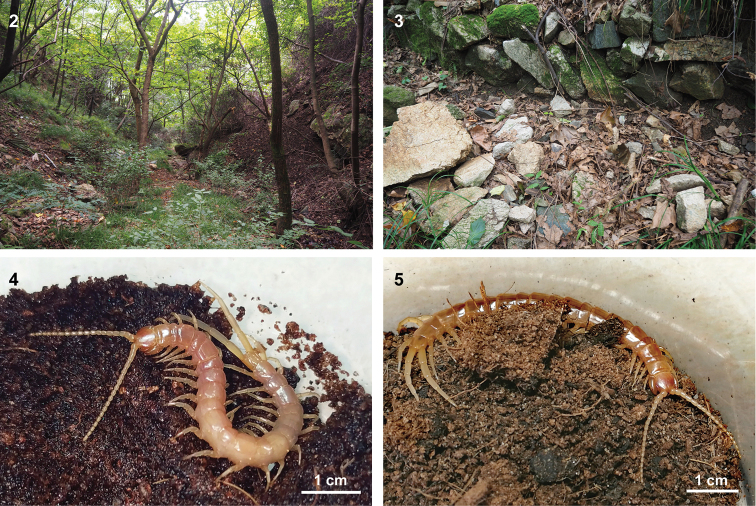
Habitat of *Mimops orientalis***2, 3** Habitat at locality of *M. orientalis* in Taiping National Forest Park, Hu county, Shaanxi, China **4, 5** living specimens of *M. orientalis*.

Specimens of *M. orientalis* were collected from two locations on Qinling Mountain. We captured all of our new material at midday (11:00–13:00), always in forest edges near a river and under microhabitat refuges (rocks, bushes, leaf litter, and garbage). Two adult specimens were hiding under stones coated with moss, and another adult was encountered under some garbage containing rainwater. Most juveniles were encountered under small stones in bushes or moving over leaf litter in a fragmented patch of *Liquidambar formosana* Hance. Natural vegetation in the surrounding areas is deciduous forest, composed mainly of *Juglans regia* L., *Cotinus coggygria* Scop., and *L. formosana* Hance. Vegetative cover was ornamented by *Urtica* spp., *Viola* spp., *Cyperus iria* L., and *Oplismenus undulatifolius* (Arduino) Beauv (Figs 2, 3). We fed captive centipedes with mealworms, fish moths, and cockroaches, the latter two typically occurring in the centipedes’ microhabitat. The centipedes became active at approximately 20:00 most nights and would quickly escape to shelter when disturbed by noise.

#### Live colouration.

Cephalic plate brownish red to orange-red, more deeply coloured in the anterior part. Coxosternites, forcipules, and tergites pale yellow to pale brown, ultimate segment and ultimate legs pale orange. Antennae, all sternites, and dorsal aspects of the legs light yellow. The live colour of a specimen from Shaanxi is slightly different from others. Its ultimate leg prefemur was nearly brown, which is more deeply coloured than that of the femur and tibia. The juvenile individual was uniformly light yellow on the cephalic plate, antennae, tergites, sternites, and all legs (Figs 4, 5).

#### Redescription.

Lewis (2006) examined and described the holotype of *M. orientalis*. The majority of features of the newly collected specimens are in agreement with the holotype, but with some differences.

**Figures 6–9.**
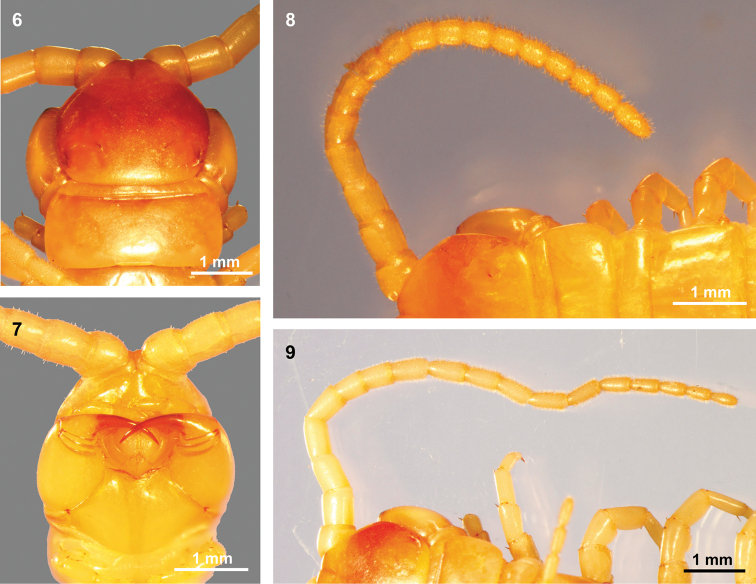
Head of *Mimops orientalis* Kraepelin, 1903 **6, 7** Head and tergite 1 and 2, dorsal and ventral views **8, 9** dorsal views of the right antenna.

Adult length 42–56 mm. Cephalic plate smooth, about as long as it is wide, very finely punctate with the posterior margin overlying tergite 1. Cephalic plate lacks paramedian sulci in adults (two sulci in juvenile). A pale area instead of lateral ocelli at the base of each antenna (Lewis [2006] stated that is a single ocellus, whereas Kraepelin [1903] treated this as an eyespot). The animals show no response when the pale areas are illuminated with searchlights. Antenna extend to the posterior end of tergite 8, usually with 18 articles (holotype with 17 articles on the right antenna). Articles approximately 1.20–1.42 times as long as they are wide (based on article 4). Amount of hair on the antenna gradually increases, with the presence of glabrous articles variable (Lewis [2006] stated that the basal six articles were glabrous, while Kraepelin (1903) stated that the basal seven articles were glabrous). In our collected specimens, basal articles 4 and 5 were dorsally nearly glabrous (Figs 6–9), and articles 5–7 (Fig. 8, specimen CMMI 20190908003) or 6 and 7 (Fig. 9, specimen CMMI 20190908004) had sparse hair; articles 8–18 were densely hirsute.

**Figures 10–15.**
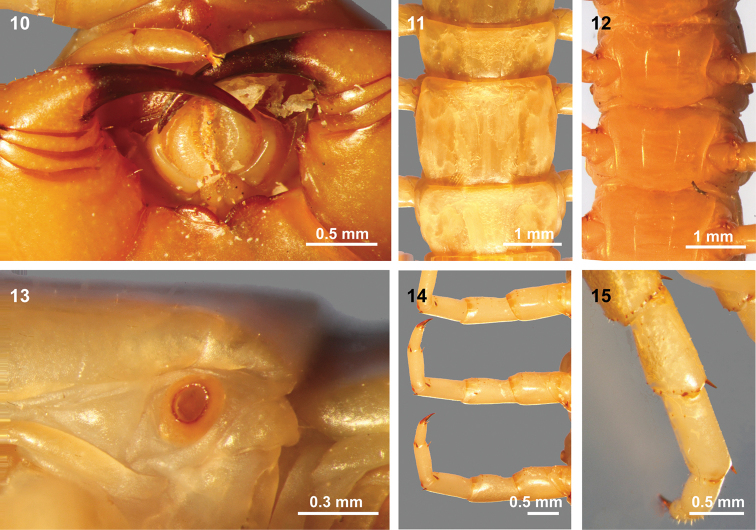
**10** Coxosternite and forcipules of *Mimops orientalis***11–15** Trunk segment features of *Mimops orientalis*. **11** Tergites 11–13, showing paramedian sutures and spiracle **12** sternites 5–7, showing sutures **13** spiracle on segment 5 **14** ventral view of legs 5–7 (right) **15** dorsal view of leg 3 (right).

Tooth plates are short but wide, ridged and have approximately 25 ridges on each side and a prominent seta behind the anterior margin (Fig. 10). Forcipular trochanteroprefemur processes are very short, apex truncated, and with a long and acute process spine. Forcipular medial tibia and femur also have a prominent acute spine. Articles 1–3 of the second maxillary telopodite show hair and apical claw form a spine with long comb hair (Fig. 10).

Tergite 1 with anterior transverse (ring) sulcus (Fig. 6). Tergites 2–20 with complete paramedian sutures. Two longitudinal sulci lateral to the paramedian sutures on the five posterior tergites except the ultimate tergite. Tergites 2–20 without margination. Posterior part of the ultimate leg-bearing segment with complete margination. Sternites with complete paramedian sulci from 3 to 19, almost complete on sulcus 2. Nine round spiracles are present with one each on segments 3, 5, 8, 10, 12, 14, 16, 18, and 20. The spiracles protrude out of the segments (Figs 11, 12), and are cup-shaped, with a simple structure, and without humps (Fig. 13) (Lewis [2006] stated that the spiracles were filled with humps).

All legs with two-segmented tarsi. Two tibial spurs on legs 1–18, 19 with one, 20 without. Legs 1–20 each with two tarsal spurs (Fig. 14). Legs 1–5 with one outside femur spur dorsally, legs 1–3 also with one prefemur spur dorsally (Fig. 15). Legs 1–14 with very few spines ventrally on the prefemur and femur and a distal transverse row dorsomedially on the prefemur, femur, and tibia (Fig. 14). Legs 15 and 16 with few spines ventrally on the prefemur and sparse spines ventrally on the femur. Legs 17–19 with prefemur, femur, and tibia thickly spined dorsally, medially, and ventrally, the spines on prefemur gradually increase from legs 17–19. Leg 20 with prefemur and femur thickly spined on all surfaces, tibia on all but the medial surface and tarsus 1 spined dorsally and medially.

**Figures 16–18.**
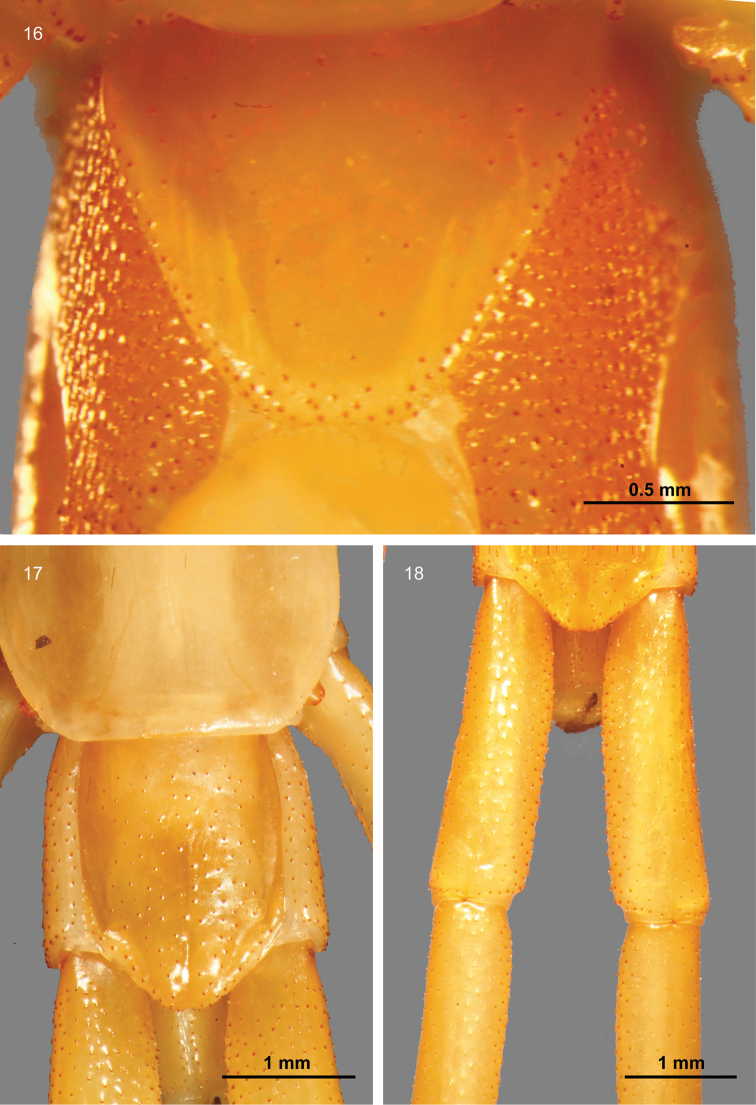
Ultimate segment and ultimate legs of *Mimops orientalis***16** Ventral view of the ultimate segment **17** dorsal view of the ultimate segment **18** dorsal view of ultimate legs.

**Figures 19, 20.**
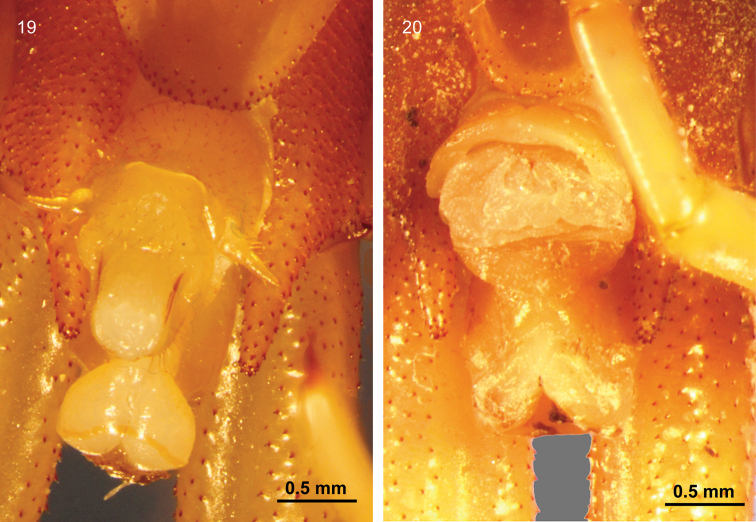
Genital segments of *Mimops orientalis***19** Male specimen **20** female specimen.

Tergite 21 with small spines and a narrow posterior median depression (Fig. 17). Sternite 21 with sides converging posteriorly, without depression. Central zone of sternite 21 with sporadic small spines dorsal and ventral and peripheral zone with two to four rows of small spines (Fig. 16, 19). Coxopleuron with an oval pore field of many small pores and small scattered spines (Fig. 19). The coxopleural process was moderately long and digitiform, with small spines (Figs 19, 20). The prefemur and femur of the ultimate legs (Fig. 18) are covered with numerous small spines dorsally, medially, and ventrally, without grooves or strips (Lewis [2006] stated with the presence of a strip on the median ventral). Tibia spined on all but the median ventral and medial surfaces, and tarsus 1 has a few dorsomedial spines. Pretarsal accessory spurs are absent.

Genital segments well developed, reaching the distance between the posterior margin of sternite 21 and the distal part of the coxopleural process. In males, sternite of genital segment 1 round, with short setae ventrally, genital segment 2 round and convex, also with short setae ventrally. Penis columnar, with long setae dorsally. Gonopod present in males with seven or eight long setae (Fig. 19). Anal valve well developed, composite by two hemispheres, with a yellow strip near the posterior margin. Genital segments short in female, posterior margin of sternite of genital segment 1 with several spines. Anal valve composed of two hemispheres, with a cavity in the centre.

#### Conservation status.

Although this species was found from two localities separated by 400 km, there is not yet enough information about the distribution, abundance, or threats to this species, and so further surveys are needed; we consider it Data Deficient for now (IUCN 2020).

#### Molecular analyses.

We obtained 887 bp sequences of COI, 537 bp of 16S and 992 bp of 28S of *M. orientalis*. The complete matrix included sequences from 44 centipede species (Table 1), which consists of all five families of Scolopendromorpha. *Theatops chuanensis* Di et al., 2010, *Cryptops* sp., *Scolopendra mutilans* L. Koch, and *Scolopocryptops nigrimaculatus* Song, Song & Zhu, 2004 were also sequenced and subjected to phylogenetic analysis. These species represent the most common species of the families Plutoniumidae, Cryptopidae, Scolopendridae, and Scolopocryptopidae, respectively, in China. For the class Chilopoda, which is commonly divided into five orders, namely Scolopendromorpha, Lithobiomorpha, Scutigeromorpha, Craterostigmomorpha, and Geophilomorpha, four representative species, *Scutigera coleoptrata* (Scutigeromorpha), *Craterostigmus tasmanianus* (Craterostigmomorpha), *Lithobius forficatus* (Lithobiomorpha), and *Bothriogaster signata* (Geophilomorpha) were selected as outgroups to assess the phylogenetic of *M. orientalis* as well as Mimopidae.

**Figures 21, 22.**
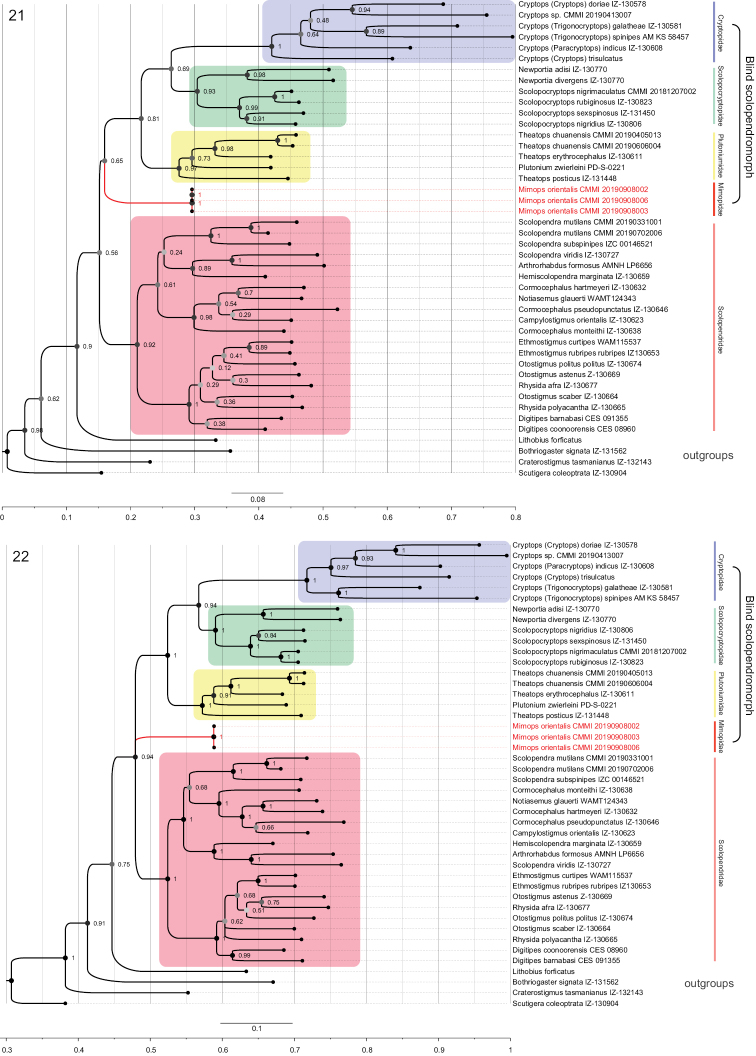
Phylogenetic trees obtained from the COI, 16S, and 28S sequences **21**ML tree from the analysis of the concatenated genes **22** tree from the Bayesian analysis of all genes combined. Numbers at the nodes are bootstrap percentages obtained from the ML analyses and posterior probabilities obtained from BI.

The results of the phylogenetic analysis are presented in Figures 21 and 22. Bayesian inference (BI) and maximum likelihood (ML) analyses yielded trees based on the combined alignments of COI + 16S + 28S, which demonstrated essentially consistent topologies. All Scolopendromorpha species used in this study could be considered as two main clades, the ocellate scolopendromorphs and the blind scolopendromorphs, both well supported in the ML tree. Mimopidae was positioned as sister group to the other families of blind scolopendromorphs. However, Bayesian inference analyses identified Scolopendromorpha species as belonging to three clades, the ocellate scolopendromorphs, the blind scolopendromorphs sensu Pocock, 1896 (comprising Plutoniumidae, Cryptopidae, and Scolopocryptopidae), and the monophyletic family Mimopidae with quite strong support (PP 0.94). Both methods obtained from the molecular data place Scolopendridae as a monophyletic family, comprising two subfamilies, Scolopendrinae and Otostigminae. Likewise, Scolopocryptopidae comprises two subfamilies, Scolopocryptopinae and Newportiinae. Cryptopidae, and Scolopocryptopidae are sister groups together with Plutoniumidae in the molecular analysis.

**Table 1.** List of the 44 selected sequences obtained from this study GenBank nucleotide database used in phylogenetic analyses.

Taxonomy	Species	Voucher number	Taxon locality	GenBank accession number			Reference
				COI	16S	28S	
Mimopidae	*Mimops orientalis*	CMMI 20190908002	Shaanxi, China	MT093838	MT084401	MT084368	This study
	*Mimops orientalis*	CMMI 20190908003	Shaanxi, China	MT093839	MT084402	MT084369	This study
	*Mimops orientalis*	CMMI 20190908006	Shaanxi, China	MT093840	MT084403	MT084370	This study
Cryptopidae	Cryptops (Cryptops) trisulcatus		Italy	HQ402544	HQ402493	AF000783	1
	Cryptops (Cryptops) doriae	IZ-130578	Thailand	KF676500	KF676458	KF676354	1
	Cryptops (Trigonocryptops) galatheae	IZ-130581	Argentina	KF676501	KF676459	KF676355	1
	Cryptops (Trigonocryptops) spinipes	AM KS 58457	Australia	AY288743	AY288724	AY288709	1
	Cryptops (Paracryptops) indicus	IZ-130608	Vietnam	KF676505	KF676463	KF676357	1
	*Cryptops* sp.	CMMI 20190413007	Liuan, China	MT093841	MT084404	MT084371	This study
Plutoniumidae	*Theatops erythrocephalus*	IZ-130611	Portugal	HM453313	HM453222	AF000784	1
	*Theatops posticus*	IZ-131448	USA	AY288746	AY288727	–	1
	*Theatops chuanensis*	CMMI 20190405013	Hunan, China	MT093842	MT084405	MT084372	This study
	*Theatops chuanensis*	CMMI 20190606004	Gansu, China	MT093843	MT084406	MT084373	This study
	*Plutonium zwierleini*	PD-S-0221	Italy	LN890292	LN890289	LN890291	
Scolopocryptopidae	*Newportia divergens*	IZ-130770	Mexico	JX422667	JX422690	KF676359	1
	*Newportia adisi*	IZ-130770	Brazil	KF676506	KF676465	JX422586	1
	*Scolopocryptops nigridius*	IZ-130806	USA	JX422680	JX422704	JX422594	1
	*Scolopocryptops rubiginosus*	IZ-130823	Taiwan	JX422682	JX422706	–	1
	*Scolopocryptops nigrimaculatus*	CMMI 20181207002	Hangzhou, China	MT093844	MT084407	–	This study
	*Scolopocryptops sexspinosus*	IZ-131450	USA	AY288745	AY288726	AY288710	1
Scolopendridae	*Scolopendra subspinipes*	IZC 00146521	Martinique	HQ402554	HQ402502	HQ402538	1
	*Scolopendra mutilans*	CMMI 20190331001	Hubei, China	MT093845	MT084408	MT084374	This study
	*Scolopendra mutilans*	CMMI 20190702006	Zhejiang, China	MT093846	MT084409	MT084375	This study
	*Scolopendra viridis*	IZ-130727	USA	DQ201431	DQ201425	DQ222134	1
	*Cormocephalus hartmeyeri*	IZ-130632	Australia	KF676531	KF676491	KF676391	1
	*Cormocephalus monteithi*	IZ-130638	Australia	DQ201430	AF370861	AF173280	1
	*Cormocephalus pseudopunctatus*	IZ-130646	South Africa	KF676534	KF676493	KF676398	1
	*Hemiscolopendra marginata*	IZ-130659	USA	HQ402548	HQ402496	–	1
Scolopendridae	*Arthrorhabdus formosus*	AMNH LP6656	South Africa	HQ402539	HQ402488	HQ402522	1
	*Campylostigmus orientalis*	IZ-130623	New Caledonia	HQ402542	HQ402491	KF676404	1
	*Notiasemus glauerti*	WAMT124343	Australia	KF676539	KF676498	KF676405	1
	*Otostigmus astenus*	IZ-130669	Fiji	HM453312	HM453221	HQ402532	1
	*Otostigmus politus politus*	IZ-130674	China	KF676512	KF676470	KF676368	1
	*Otostigmus scaber*	IZ-130664	Taiwan	KF676513	KF676471	KF676369	1
	*Digitipes barnabasi*	CES 091355	India	JX531905	JX531775	JX531826	2
	*Digitipes coonoorensis*	CES 08960	India	JX531850	JX531720	JX531793	2
	*Ethmostigmus curtipes*	WAM115537	Australia	KF676515	KF676474	KF676372	1
	*Ethmostigmus rubripes rubripes*	IZ130653	Australia	KF676542	KF676475	KF676373	1
	*Rhysida afra*	IZ-130677	South Africa	HQ402552	HQ402500	HQ402536	1
	*Rhysida polyacantha*	IZ-130665	Australia	KF676518	KF676476	KF676376	1
Lithobiomorpha	*Lithobius forficatus*	–	–	AJ270997	AJ270997	EF199984	3
Craterostigmomorpha	*Craterostigmus tasmanianus*	IZ-132143	Australia	EU024611	EU024597	HM453265	1
Geophilomorpha	*Bothriogaster signata*	IZ-131562	Greece	AY288749	AY288730	HM453290	1
Scutigeromorpha	*Scutigera coleoptrata*	IZ-130904	South Africa	DQ222170	DQ222156	EF199983	1

1 = Vahtera et al. 2013; 2 = Joshi and Karanth 2012; 3 = Hwang et al. 2001.

## Discussion

**Figures 23–27.**
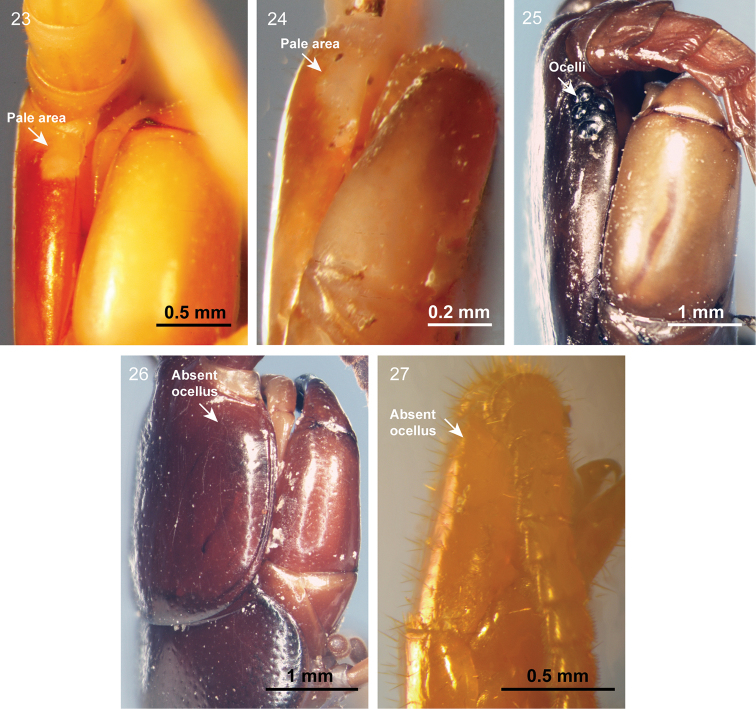
Ocelli characteristics of Scolopendromorpha**23***Mimops orientalis***24***Theatops chuanensis***25***Scolopendra mutilans***26***Scolopocryptops nigrimaculatus***27***Cryptops* sp.

The taxonomic position of *Mimops* has been controversial for many years. Attems (1930) divided scolopendromorphs into blind scolopendromorphs and ocellate scolopendromorphs, the latter containing only a single family, Scolopendridae, whereas the former includes three eye-less scolopendromorph families (Cryptopidae, Plutoniumidae, and Scolopocryptopidae), hence, the name blind clade. The genus *Mimops* was first classified into the subfamily Cryptopinae of the family Cryptopidae by Attems (1930) and then removed from Cryptopinae in a cladistic analysis by Schileyko and Pavlinov (1997). Shelley (2002) moved *Mimops* to the Scolopendridae, and subsequently Lewis (2006) restudied the holotype and placed it in a new family, Mimopidae. Most recently, Edgecombe and Giribet (2019) also treated *Mimops* as an ocellate scolopendromorph in the monotypic family Mimopidae. A critical controversy focused on the presence or absence of ocelli in *M. orientalis*. Attems (1930) and Schileyko treated the “Augenfleck” not as a real eye and placed the species in the blind clade, whereas Lewis, Shelley, and Edgecombe considered it as a single ocellus. With careful inspection, we found that *M. orientalis* has two round pale areas, rather than ocelli, at the base of the antenna. The pale area of *M. orientalis* is identical to that of *Theatops chuanensis* of the family Plutoniumidae, but absent in Scolopocryptopidae (Figs 23–27). However, ocelli are not an arbitrary family characteristic in scolopendromorphs. *Tonkinodentus* Schileyko, 1992, an eye-less scolopendrid-like genus, was unequivocally supported inside Scolopendridae by morphological and molecular data (Schileyko et al. 2019). Three recently described scolopendrid centipedes, *Cormocephalus sagmus*Edgecombe et al. 2019, *C. pyropygus*Edgecombe et al. 2019, and *C. delta*Edgecombe et al. 2019, form a new blind species group in the genus *Cormocephalus* Newport, 1844 which otherwise presents four ocelli. *Mimops* also shares many scolopendrid-like characteristics, but distinct differences from *Tonkinodentus* and *Cormocephalus* imply that eye loss may have occurred several times in the evolution of Scolopendromorpha.

*Mimops orientalis* also possesses several unusual characteristics among Plutoniumidae, Cryptopidae, Scolopocryptopidae, and Scolopendridae. It shares many characteristics with scolopendrid centipedes, including a smooth cephalic plate without setae or spines, several glabrous basal antennal articles, the presence of sternite and tergite paramedian sutures, the presence of spines on the prefemora of the ultimate legs, and an anterior transverse sulcus on tergite 1. These characteristics render it similar to *Scolopendra* species, particularly many New World varieties. However, *M. orientalis* lacks lateral ocelli and instead possesses a pale area, which is only present in plutoniumids, and the spines on the ultimate legs are very small, which is similar to some *Trigonocryptops* species, e.g., Cryptops (Trigonocryptops) camoowealensis Edgecombe, 2006. This renders the taxonomic position of *Mimops* puzzling.

In this study, we confirmed Mimopidae as a valid family based on both morphological and molecular data. We also place *Mimops* as the basal taxa of scolopendromorphs for the following reasons: 1) *M. orientalis* is resolved as monophyletic both in maximum likelihood and Bayesian inference analyses under all parameter sets that were explored; 2) the ocelli of *Mimops* are regressed and have left two pale areas, whereas the outgroups Lithobiomorpha, Scutigeromorpha, and Craterostigmomorpha all have ocelli (or a compound eye with ommatidia in the case of Scutigeromorpha), indicating that ocelli may be a plesiomorphic characteristic; 3) *Mimops* bears 21 trunk segments, whereas 21 to 23 segments is an unreversed apomorphy of Scolopocryptopidae; 4) the long and acute spines on the forcipular trochanteroprefemoras of *Mimops* may be a primitive characteristic of trochanteroprefemur process; and 5) the small homologous spines on the ultimate legs, coxopleural process and ultimate sternites seem to be an intermediate state between setae and spines. However, although BI and ML analyses both support the validity of Mimopidae, the precise position of *Mimops* is not delimited due to the unresolved trichotomy in BI tree. Further research based on complete mitochondrial genome or transcriptomic data may help to clarify phylogenetic relationships among Mimopidae and other families.

## Supplementary Material

XML Treatment for Mimops orientalis
